# In-Hospital Use of Long-Acting Injectable Antipsychotics and Readmission Risk in Patients With First-Admission Schizophrenia in Taiwan

**DOI:** 10.1001/jamanetworkopen.2024.17006

**Published:** 2024-06-17

**Authors:** Wei Chen, Chi-Shin Wu, Chen-Chung Liu, Po-Hsiu Kuo, Hung-Yu Chan, Yi-Hsuan Lin, Yu-Chu Ella Chung, Wei J. Chen

**Affiliations:** 1 Institute of Epidemiology and Preventive Medicine, College of Public Health, National Taiwan University, Taipei, Taiwan; 2 National Center for Geriatrics and Welfare Research, National Health Research Institutes, Miaoli, Taiwan; 3 Department of Psychiatry, College of Medicine and National Taiwan University Hospital, National Taiwan University, Taipei, Taiwan; 4 Department of Public Health, College of Public Health, National Taiwan University, Taipei, Taiwan; 5 Taoyuan Psychiatric Center, Ministry of Health and Welfare, Taoyuan City, Taiwan; 6 Center for Neuropsychiatric Research, National Health Research Institutes, Miaoli, Taiwan

## Abstract

**Importance:**

Long-acting injectable antipsychotics (LAIs) can help decrease the rate of nonadherence to medications in patients with schizophrenia, but these drugs are underutilized in clinical practice, especially in Asian countries. One strategy for the early prescription of LAIs is to administer the drugs during patients’ first admission, when they have more time to absorb medication-related knowledge.

**Objective:**

To estimate the prevalence of and risk factors for in-hospital use of LAIs among first-admission patients with schizophrenia in Taiwan and to examine the association of early discontinuation with readmission risk among patients receiving LAIs.

**Design, Setting, and Participants:**

This cohort study included data from a claims database for patients with a first admission for schizophrenia at psychiatric wards in Taiwan from 2004 to 2017. Eligible patients were diagnosed with schizophrenia or schizoaffective disorder at discharge and aged between 15 and 64 years. Data analysis was performed from April to September 2022.

**Exposure:**

In-hospital use of LAIs with or without early discontinuation.

**Main Outcome and Measures:**

Readmission for any psychotic disorder following discharge from first admission, with risk estimated via multivariable survival regression analysis, including the Cox proportional hazards (CPH) model and accelerated failure time (AFT) model.

**Results:**

Of the 56 211 patients with a first admission for schizophrenia (mean [SD] age, 38.1 [12.1] years; 29 387 men [52.3%]), 46 875 (83.4%) did not receive any LAIs during admission, 5665 (10.1%) received LAIs with early discontinuation, and 3671 (6.5%) received LAIs without early discontinuation. The prevalence of receiving LAIs increased by 4%, from 15.3% (3863 of 25 251 patients) to 19.3% (3013 of 15 608 patients) between 2004-2008 and 2013-2017. After controlling for sex, year, prior antipsychotic use, age at first admission, and length of stay, the CPH regression analysis revealed that the readmission risk increased among patients receiving LAIs with early discontinuation (adjusted hazard ratio [aHR], 1.25; 95% CI, 1.21-1.30) but decreased among patients receiving LAIs without early discontinuation (aHR, 0.88; 95% CI, 0.84-0.92) compared with patients not receiving LAIs. Results remained similar for the AFT model.

**Conclusions and Relevance:**

The incidence of in-hospital use of LAIs among patients with a first admission for schizophrenia has remained low. In this study, early discontinuation of LAIs was associated with readmission risk—specifically, early discontinuation with a higher risk while the lack of early discontinuation with a lower risk compared with treatment with oral antipsychotics alone—which suggests our results have implications for improving the efficacy of LAI administration among patients with a first admission for schizophrenia.

## Key Points

**Question:**

Is administering long-acting injectable antipsychotics (LAIs) to first-admission patients with schizophrenia associated with reduced risk of readmission?

**Findings:**

In this cohort study of 56 211 patients with first-admission schizophrenia, the readmission risk increased by 22% to 25% among patients receiving LAIs with early discontinuation but decreased by 12% to 13% among patients receiving LAIs without early discontinuation compared with patients not receiving LAIs.

**Meaning:**

These results suggest that early discontinuation of in-hospital use of LAIs is associated with increased rates of readmission; our results have implications for improving the efficacy of LAI administration among first-admission patients with schizophrenia.

## Teaser

This cohort study of Taiwanese patients with a first admission for schizophrenia examines the prevalence of in-hospital long-acting injectable antipsychotic (LAI) use and assesses whether LAIs are associated with subsequent readmission.

## Introduction

Antipsychotics have long been the preferred treatment for schizophrenia, with oral antipsychotics being the most commonly prescribed form.^1^ However, nonadherence to oral medications is a common cause of relapse, rehospitalization, and poor outcomes.^2,3^ Alternatively, long-acting injectable antipsychotics (LAIs) can be administered at an interval of 2 weeks to 1 or more months and are associated with greater medication adherence^4,5,6^ but similar adverse effects^7,8^ compared with oral antipsychotics. Since LAIs tend to be prescribed for more severely ill patients, their beneficial efficacy could be detected only in clinical trials that enrolled patients at high risk for nonadherence^8^ and cohort studies that adopted a within-individual approach in analysis.^9,10^ Both randomized clinical trials^11,12,13,14^ and cohort studies^9,10,15,16^ of patients with early phase or first-episode schizophrenia have also indicated that LAIs have benefits in preventing relapses and hospitalizations.

Despite the evidence supporting the use of LAIs, these drugs remain largely underutilized in clinical practice, especially in Asian countries.^16,17,18^ Some of the reasons include clinicians’ unfamiliarity with LAIs and insufficient time to explain the rationale for prescribing LAIs to patients or their family members.^19,20^ Furthermore, a 2019 systematic review revealed that even for second-generation LAIs, the discontinuation rate before 36 weeks was approximately 50%.^21^ One potential strategy for the early prescription of LAIs is to administer the drugs during patients’ first admission, when they can receive peer support and have more time to absorb medication-related knowledge.^16^ Although previous studies have investigated the in-hospital use of LAIs in a small number of hospitals in the US^22,23,24^ or Switzerland,^25^ few studies have examined the administration of LAIs to first-admission patients with schizophrenia nationwide.

To fill this gap in the research, we examined the national cohorts of first-admission patients with schizophrenia established by the National Health Insurance Research Database (NHIRD)^26^ from 2004 to 2017 in Taiwan. This study aimed to (1) estimate the prevalence of in-hospital use of LAIs in first-admission patients with schizophrenia and the occurrence of early discontinuation of LAIs among those receiving LAIs; (2) examine the correlates of in-hospital use of LAIs; and (3) compare the readmission risk among patients who received LAIs with or without early discontinuation vs those not receiving LAIs after adjusting for potential confounders.

## Methods

### Data Source

The data used in this study were extracted from the NHIRD for Psychiatric Inpatients, a subdatabase of NHIRD that had a high coverage rate of Taiwan’s residents (97% in 2001 and above 99.9% in 2014).^26^ Although employed noncitizens can join NHI, their medical utilization only accounts for 0.4% in 2010 according to the statistics by National Health Insurance Administration. Once the cohort membership of a patient was established, all the patient’s outpatient and inpatient claims during the study period were retrieved. This project was approved by the Research Ethics Committee of the National Taiwan University Hospital. Because the data of the NHIRD were deidentified before being accessed by researchers, a waiver of informed consent was granted. This study followed the Strengthening the Reporting of Observational Studies in Epidemiology (STROBE) reporting guideline for cohort studies.

### Cohort Selection

The eligible cohort dated from January 2004 to December 2017; relevant information (prior antipsychotic treatment history) was collected retrospectively up to 3 years before enrolling in the study. In the derivation of the data set, we first selected patients who were admitted to psychiatric wards for the first time between January 1, 2001, and December 31, 2017, and who were diagnosed with schizophrenia or schizoaffective disorder at discharge (*International Classification of Diseases, Ninth Revision, Clinical Modification *[*ICD-9-CM*] code 295; *ICD-10-CM* codes F20 and F25). The exclusion criteria were as follows: (1) aged younger than 15 years or older than 64 years (2900 patients) owing to the concern that patients with such an early first-admission age might have etiologic factors rendering a poor outcome whereas patients with such an old first-admission age might have other organic conditions, (2) missing sex information in the database (858 patients), and (3) died during the first admission (103 patients). More details about this selection process can be found elsewhere.^16^ The cohorts of first-admission patients from 2001 to 2003 were then excluded because less than 3 years information on prior antipsychotic treatment history proved to be inadequate (excluding 19 725 patients). We further excluded patients who had inaccurate LAI records or who were censored before the first discharge (50 patients). Because the NHIRD did not provide information on race or ethnicity, we did not consider them as a study variable.

### Exposure

The main concern of this study was whether a patient was prescribed any LAI during their first admission. All the LAIs available during the study period are displayed in eTable 1 in Supplement 1. Between 2004 and 2017, first-generation LAIs predominated over second-generation LAIs until 2015, when the 2 converged; subsequently, the trend reversed (eFigure 1 in Supplement 1).

In addition, we characterized whether a patient’s use of LAIs was associated with early all-cause discontinuation. Following the definition of all-cause treatment discontinuation as “the period between LAI antipsychotic prescriptions that exceeded the allowable gap,”^27^ we set this allowable gap as 30 days given the availability of LAIs during the study period. With the intention of identifying the earliest sign of nonadherence, we classified a patient as having early discontinuation if there were zero or 1 continuous LAI prescriptions before all-cause treatment discontinuation occurred.

Hence, patients were divided into 3 groups according to their 3-level exposure status: (1) received LAIs without early discontinuation, (2) received LAIs with early discontinuation, and (3) did not receive any LAIs, ie, treatment with oral antipsychotics alone. The flowchart of patient selection and exposure classification is displayed in eFigure 2 in Supplement 1.

### Outcome

The outcome of this study was readmission for any psychotic disorder. The diagnostic categories of psychotic disorders included in this study were the same as those used in a previous study.^16^

### Previous Antipsychotic Treatment

We collected information about patients’ antipsychotic prescription experience during the 3 years prior to their first admission date. Any medication with an ATC code beginning with N05A was identified as an antipsychotic. The decision of whether a patient was prescribed antipsychotics was made separately for each year. Based on each year’s antipsychotic prescription during the 3-year period before the first admission, we categorized patients into 4 groups: (1) 3 years with prescription, ie, received antipsychotic prescriptions in each year; (2) recent yearlong discontinuation, ie, yearlong discontinuation leading to admission but with antipsychotic prescription in earlier years; (3) less than 2 years with prescription, ie, with antipsychotic prescription in the year before the first admission but without antipsychotic prescription in at least 1 earlier year; and (4) antipsychotic-free, ie, no antipsychotic prescription during the 3-year period (eTable 2 in Supplement 1).

### Statistical Analysis

The Kruskal-Wallis test was used to compare continuous variables, and the χ^2^ test was used to compare categorical variables. Readmission risks were estimated using the Kaplan-Meier estimator of survival analysis with the first psychotic readmission as the endpoint event or censored (including death, reaching age 65 years old, follow-up for more than 4 years or study end). Log-rank tests were used to compare different Kaplan-Meier survival curves. Cox proportional hazards (CPH) regression analysis was subsequently performed to adjust for multiple covariates. If the assumption of proportional hazards was violated, we further performed exponential accelerated failure time (AFT) regression analysis to adjust for these covariates. In the AFT model, a longer time to event indicates a lower risk, and the exponential of the corresponding coefficient results in a ratio of the mean survival time of the exposed group vs the reference group greater than 1.

For sensitivity analysis, we used a different definition of early discontinuation for LAIs and conducted a multivariable CPH regression model by removing antipsychotic use experience during the 3 years prior to the first admission from the covariate set to assess its potential collider bias. All the analyses were performed in 2022 using SAS software version 9.4 (SAS Institute, Inc). The point estimates and 95% CIs are presented. A 2-tailed *P* value less than .05 was considered to indicate statistical significance.

## Results

Of the 56 211 patients with a first admission for schizophrenia, 29 387 (52.3%) were male, most of them were admitted during the period of 2004-2008 (25 251 [44.9%]) and antipsychotic-free in the 3-year period prior to admission (26 965 [48.0%]), and had a mean (SD) age of 38.1 (12.1) years and a mean (SD) length of stay of 36.3 (29.9) days (Table 1). Pertaining to their use of LAIs during the first admission, 46 875 (83.4%) did not receive any LAIs during the first admission, 5665 (10.1%) received LAIs with early discontinuation, and 3671 (6.5%) received LAIs without early discontinuation. In terms of conditional percentage (ie, the percentage among first-admission patients who received LAIs), 60.7% had early discontinuation, and 39.3% did not. The distributions of 3-level exposures (no LAIs, LAIs with early discontinuation, and LAIs without early discontinuation) differed between periods of admission (χ^2^ = 207.59, *df* = 4; *P* < .001) and between periods of prior antipsychotic use (χ^2^ = 260.73, *df* = 6; *P* < .001). For example, among the 4 groups classified by prior antipsychotic use, the group with 3 years of prescription had the highest proportion of patients receiving LAIs without early discontinuation (1115 of 12 062 patients [9.3%]), whereas the group with no prescriptions had the lowest proportion of patients receiving LAIs without early discontinuation (1482 of 26 965 patients [5.5%]). The mean age at first admission (Kruskal-Wallis test χ^2^ = 240.34, *df* = 2; *P* < .001) and mean length of stay at first admission (Kruskal-Wallis test χ^2^ = 915.31, *df* = 2; *P* < .001) were also associated with exposure status. For example, patients not receiving LAIs were the youngest at first admission (mean [SD] age: not receiving LAI, 37.7 [12.2] years vs receiving LAI with early discontinuation, 39.8 [11.6] years) and had the shortest length of stay at first admission (mean [SD] length of stay: not receiving LAI, 34.9 [28.6] days vs receiving LAI with early discontinuation, 46.5 [40.1] days).

**Table 1.** Sociodemographic and Clinical Characteristics of First-Admission Patients With Schizophrenia Between 2004 and 2017 in Taiwan According to Experience Receiving Long-Acting Injectable Antipsychotics (LAIs)

Characteristic	First-admission patients, No. (%)	Use of LAIs during the first-admission					*P* value, group comparison
		No LAIs, No. (%)	LAIs with early discontinuation		LAIs without early discontinuation		
			No. (%)	Conditional %	No. (%)	Conditional %	
Total	56 211	46 875 (83.4)	5665 (10.1)	60.7	3671 (6.5)	39.3	
Sex							
Male	29 387 (52.3)	24 420 (83.1)	3047 (10.4)	61.3	1920 (6.5)	38.7	.06^c^
Female	26 824 (47.7)	22 455 (83.7)	2618 (9.8)	59.9	1751 (6.5)	40.1	
Year of admission							
2004-2008	25 251 (44.9)	21 388 (84.7)	2551 (10.1)	60.0	1312 (5.2)	34.0	<.001^c^
2009-2012	15 352 (27.3)	12 892 (84.0)	1453 (9.5)	59.1	1007 (6.6)	40.9	
2013-2017	15 608 (27.8)	12 595 (80.7)	1661 (10.6)	55.1	1352 (8.7)	44.9	
Previous antipsychotics use in 3-y period prior to admission							
3 y with prescription	12 062 (21.5)	9783 (81.1)	1164 (9.7)	51.1	1115 (9.3)	48.9	<.001^c^
Recent yearlong discontinuation^a^	1772 (3.2)	1455 (82.1)	189 (10.7)	59.6	128 (7.2)	40.4	
≤2 y with prescription^b^	15 412 (27.4)	13 137 (82.2)	1329 (8.6)	58.4	946 (6.1)	41.6	
Antipsychotics-free	26 965 (48.0)	22 500 (83.4)	2983 (11.1)	66.8	1482 (5.5)	33.2	
Age, mean (SD), y	38.1 (12.1)	37.7 (12.2)	39.8 (11.6)	NA	39.7 (11.0)	NA	<.001^d^
Length of stay, mean (SD), d	36.3 (29.9)	34.9 (28.6)	46.5 (40.1)	NA	38.6 (24.2)	NA	<.001^d^

Abbreviation: NA, not applicable.

^a^ Year-long discontinuation leading to admission.

^b^ With prescription in the first year before admission.

^c^ Comparison using the χ^2^ test.

^d^ Comparison using the Kruskal-Wallis test.

Among the 9336 patients receiving LAIs, 6179 (66.2%) received first-generation LAIs, and 3157 (33.8%) received second-generation LAIs (Table 2). In terms of individual antipsychotics, the greatest proportion was for flupentixol (3323 [35.6%]), followed by risperidone (2592 [27.8%]) and haloperidol (2187 [23.4%]), with less than 10% for each of the remaining 3 drugs. The proportion of patients receiving LAIs with early discontinuation was lower for second-generation LAIs (1590 of 5665 patients [50.4%]) than for first-generation LAIs (4075 of 5665 patients [66.0%]). Among the individual types of LAIs, the proportions of patients with early discontinuation were lowest for paliperidone LAI 28 (267 of 565 patients [47.3%]) and risperidone (1323 of 2592 patients [51.0%]), whereas the corresponding percentages for the first-generation LAIs ranged from 60.4% (250 of 414 patients) for fluphenazine to 67.3% (1471 of 2187 patients) for haloperidol.

**Table 2.** Types of Long-Acting Injectable Antipsychotics (LAIs) Prescribed for First-Admission Patients With Schizophrenia Between 2004 and 2017 in Taiwan

Variable	Patients, No.			*P* value, group comparison^a^
	Total (N = 9336)	With early discontinuation (n = 5665)	Without early discontinuation (n = 3671)	
Generation of LAIs				
First-generation	6179 (66.2)	4075 (66.0)	2104 (34.1)	<.001
Second-generation	3157 (33.8)	1590 (50.4)	1567 (49.6)	
LAI of specific antipsychotics				
First-generation				<.001
Flupentixol	3323 (35.6)	2187 (65.8)	1136 (34.2)	
Haloperidol	2187 (23.4)	1471 (67.3)	716 (32.7)	
Fluphenazine	414 (4.4)	250 (60.4)	164 (39.6)	
(Zu)clopenthixol	255 (2.7)	167 (65.5)	88 (34.5)	
Second-generation				
Risperidone	2592 (27.8)	1323 (51.0)	1269 (49.0)	
Paliperidone LAI 28^b^	565 (6.1)	267 (47.3)	298 (52.7)	

^a^ Comparison using χ^2^ test.

^b^ Paliperidone LAI 28 indicates paliperidone palmitate with a dosing interval of 28 days.

### Kaplan-Meier Survival Curve for Readmission Risk

**Figure 1.**
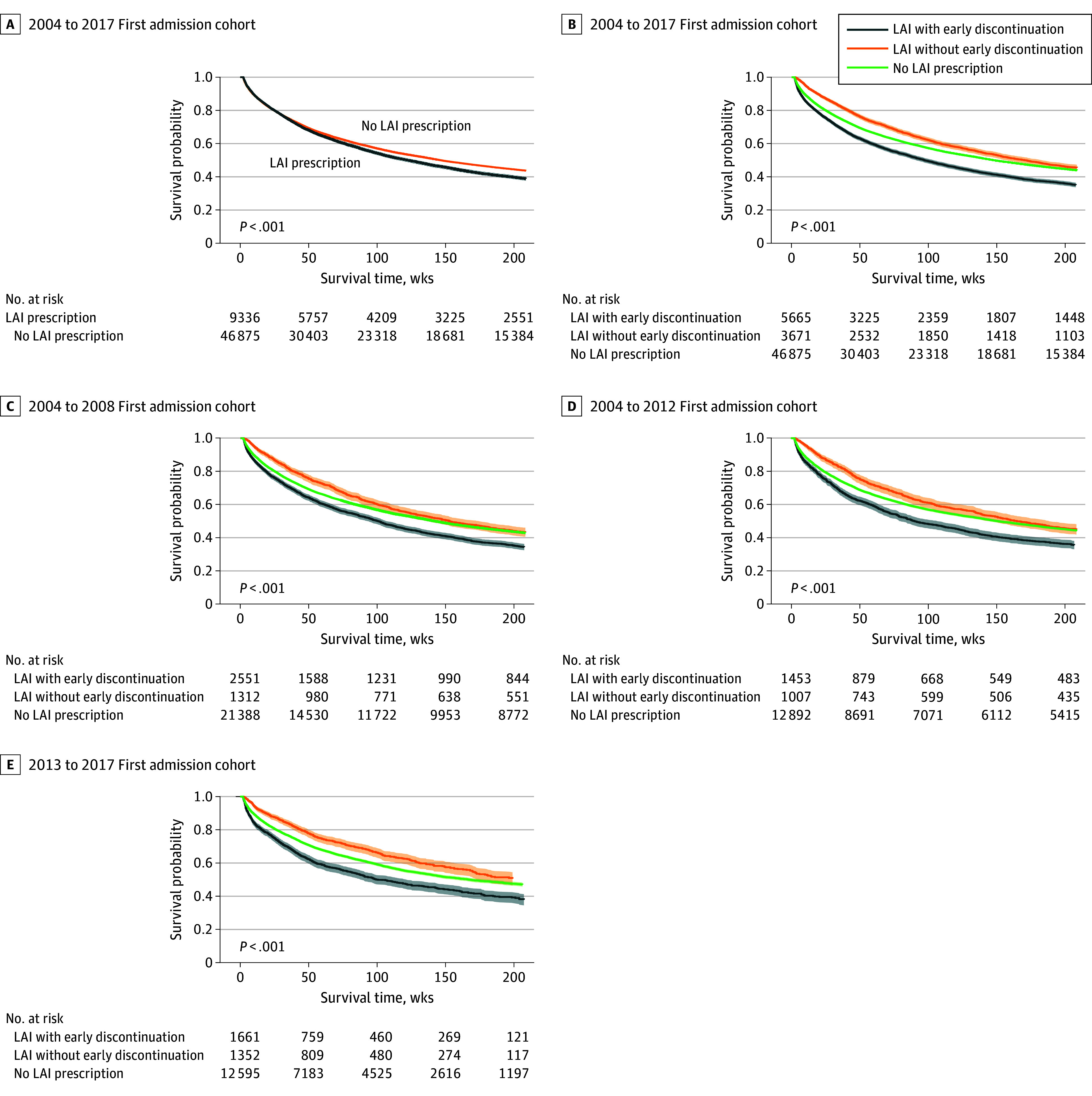
Kaplan-Meier Survival Curves of Patients With Psychotic Readmission Events in the 2004-2017 Cohort and 3 Subcohorts LAI indicates long-acting injectable antipsychotic; shaded areas represent 95% CIs. *P* values provided on the graphs were calculated by the log-rank test. A, Total cohort included 56 211 patients; B, 56 211 patients; C, 25 251 patients; D, 15 352 patients; and E, 15 608 patients.

When patients were merely dichotomized into receiving LAIs or not, their Kaplan-Meier survival curves were not significantly different (Figure 1A). When we further divided patients receiving LAIs into those who discontinued LAIs early or not, the 3 survival curves were different from each other, with the shortest median (IQR) survival time for those receiving LAIs (97.1 [25.7-not reached] weeks), the middle for those not receiving LAIs (147.6 [35.7-not reached] weeks), and the longest for those receiving LAIs without early discontinuation (170.1 [53.3-not reached] weeks) (Figure 1B).

When we divided the cohort into 3 subcohorts according to the calendar year of their first admission (ie, the 2004-2008 subcohort, 2009-2012 subcohort, and 2013-2017 subcohort), the survival curves of the 3 exposure groups remained significantly different in each subcohort (*P* < .001 for the log-rank test), in which the median (IQR) survival time from the 3 exposure groups in ascending order was 100.4 (27.1-not reached) weeks, 141.6 (35.3-not reached) weeks, and 153.1 (51.7-not reached) weeks for the 2004-2008 subcohort; 89.0 (25.4-not reached) weeks, 148.6 (34.0-not reached) weeks, and 163.3 (50.4-not reached) weeks for the 2009-2012 subcohort; and 98.9 (24.9-not reached) weeks, 165.1 (38.7-not reached) weeks, and not reached (58.0-not reached) weeks for the 2013-2017 subcohort, with the median survival time for those receiving LAI without early discontinuation longer than the current follow-up time limit (Figure 1C-1E). Hence, we included year of admission as a covariate in the subsequent multivariable survival regression analysis.

**Figure 2.**
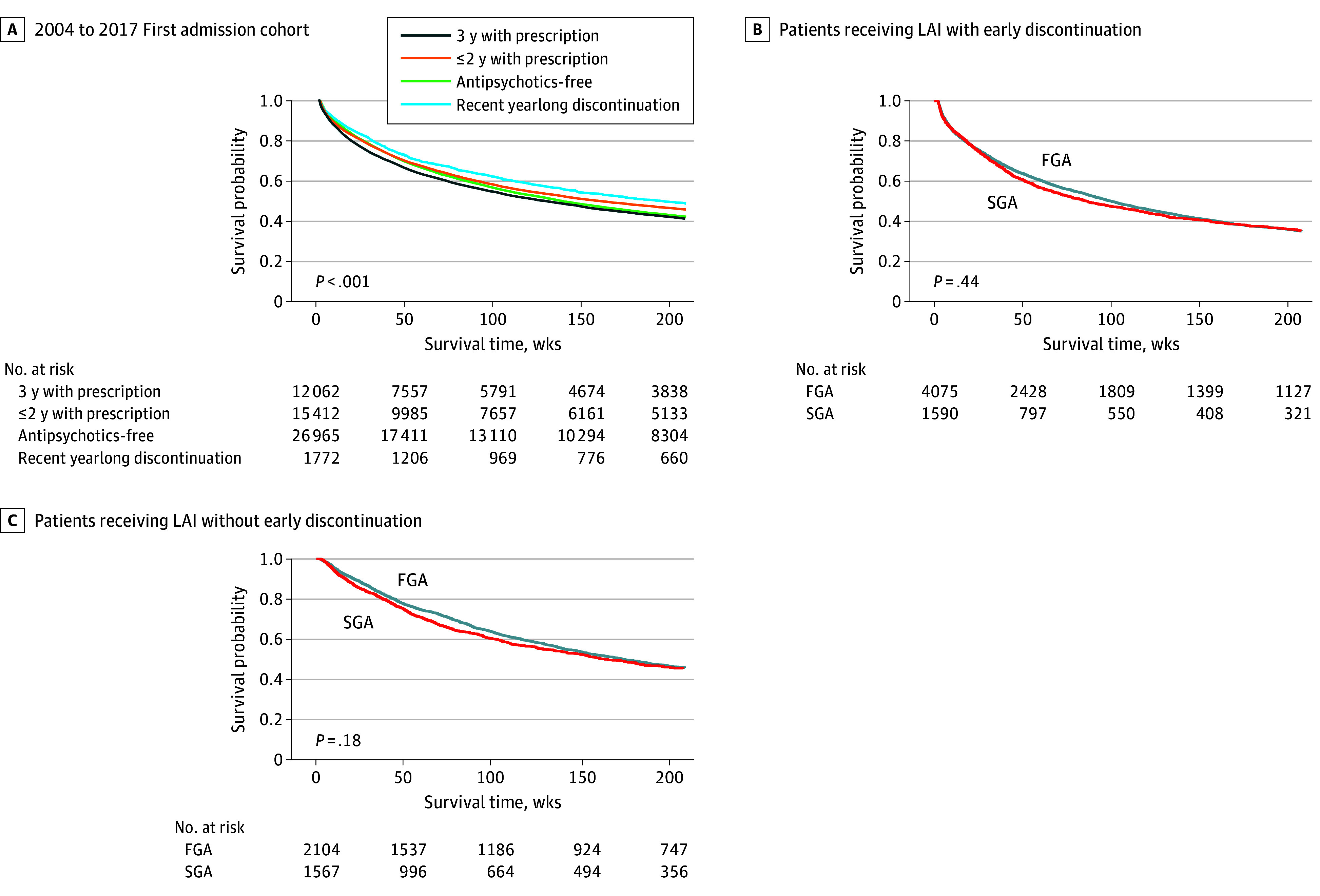
Kaplan-Meier Survival Curves of Patients at Risk of Psychotic Readmission FGA indicates first-generation antipsychotic; LAI, long-acting injectable antipsychotic; SGA, second-generation antipsychotic.

When the readmission risk was compared for patients with different prior antipsychotic use, the 3-year prescription group had the highest readmission risk, followed by the antipsychotic-free group, the group with fewer than 2 years with prescription, and the recent yearlong discontinuation group (*P* < .001 for log-rank test) (Figure 2A). Moreover, there was no significant difference between first-generation LAIs and second-generation LAIs for patients receiving LAIs with early discontinuation or patients receiving LAIs without early discontinuation (Figures 2B and 2C). Hence, the generation of LAIs was not included as a covariate in subsequent multivariable survival regression analyses.

### Multivariable Survival Regression Analysis of Readmission Risk

We then applied CPH regression analysis to assess the association of in-hospital use of LAIs with readmission (Table 3). Compared with those not receiving LAIs during the first admission, patients receiving LAI with early discontinuation had an increased risk of readmission (adjusted hazard ratio [aHR], 1.25; 95% CI, 1.21-1.30), whereas patients receiving LAI without early discontinuation had a decreased risk of readmission (aHR, 0.88; 95% CI, 0.84-0.92) after adjustment for the 4 covariates. Moreover, each of the covariates, ie, sex, year of admission, antipsychotic use experience during the 3 years prior to the first admission, age at first admission, and length of stay at the first admission, remained significantly associated with readmission risk. For the year of admission, only those patients admitted during the period of 2013-2017 had a significantly lower hazard of readmission (aHR, 0.94; 95% CI, 0.91-0.97) as compared with those admitted during the period of 2004-2008. When the interaction terms between the year of admission and the exposure group were examined, it was not significant (*df* = 4, Wald χ^2^ = 8.94; *P* = .06) and hence not included in the final model.

**Table 3.** Multivariable Survival Regression Analysis of Readmission Events in the First-Admission Patients With Schizophrenia Cohort From 2004 to 2017

Variable	Readmission risk via CPH model		Time to event via exponential AFT model	
	Adjusted hazard ratio (95% CI)	*P* value	Adjusted time ratio (95% CI)	*P* value
Intercept	NA	NA	192.1 (181.73-201.87)	<.001
Exposure groups				
No LAI prescription	1 [Reference]	[Reference]	1 [Reference]	[Reference]
LAI with early discontinuation	1.25 (1.21-1.30)	<.001	0.78 (0.75-0.81)	<.001
LAI without early discontinuation	0.88 (0.84-0.92)	<.001	1.13 (1.08-1.19)	<.001
Sex				
Women	1 [Reference]	[Reference]	1 [Reference]	[Reference]
Men	1.03 (1.01-1.05)	.02	0.97 (0.94-0.99)	.005
Year of admission				
2004-2008	1 [Reference]	[Reference]	1 [Reference]	[Reference]
2009-2012	0.99 (0.96-1.02)	.40	1.016 (0.99-1.04)	.26
2013-2017	0.94 (0.91-0.97)	<.001	0.92 (0.89-0.95)	<.001
Antipsychotics use in 3-y period prior to admission				
3 y with prescription	1 [Reference]	[Reference]	1 [Reference]	[Reference]
Recent yearlong discontinuation	0.78 (0.73-0.84)	<.001	1.30 (1.21-1.40)	<.001
≤2 y with prescription	0.87 (0.85-0.90)	<.001	1.15 (1.11-1.19)	<.001
Antipsychotic-free	0.93 (0.90-0.96)	<.001	1.08 (1.04-1.11)	<.001
Age at first admission (years)	0.996 (0.995-0.997)	<.001	1.004 (1.003-1.005)	<.001
Length of stay of first admission (weeks)	1.014 (1.012-1.017)	<.001	0.984 (0.982-0.986)	<.001

Abbreviations: AFT, accelerated failure time; CPH, Cox proportional hazards; LAI, long-acting injectable; NA, not applicable.

Because there was concern that some covariates in the Cox model did not fulfill the assumption of proportional hazards, we further applied the exponential AFT model to estimate the readmission risk (Table 3). Compared with those not receiving LAIs during the first admission, patients receiving LAI with early discontinuation had a shorter time to event (adjusted time ratio, 0.78; 95% CI, 0.75-0.81), whereas patients receiving LAI without early discontinuation had a longer time to event (adjusted time ratio, 1.13; 95% CI, 1.08-1.19). Moreover, all 5 covariates remained significantly associated with the time to event in this model.

### Sensitivity Analysis

In the first sensitivity analysis that changed the definition of early LAI discontinuation by allowing 2 or fewer continuing LAI prescriptions before all-cause treatment discontinuation occurred, the Kaplan-Meier survival curves of the 3 exposure groups remained significantly different (eFigure 3 in Supplement 1). In the second sensitivity analysis that removed antipsychotic use experience during the 3 years prior to the first admission from the covariates in the multivariable CPH regression analysis, the aHRs of the exposed groups remained almost the same (eTable 4 in Supplement 1).

## Discussion

In these national cohorts of first-admission patients with schizophrenia in Taiwan, the overall prevalence of in-hospital use of LAIs was 16.6%, including 10.1% of patients having early discontinuation and 6.5% not having early discontinuation. After controlling for sex, year of admission, prior antipsychotic use, age at first admission, and length of stay, both the CPH regression analysis and the exponential AFT regression analysis consistently revealed an increased readmission risk for patients receiving LAIs with early discontinuation but a decreased readmission risk for patients receiving LAIs without early discontinuation compared to patients not receiving LAIs. Our findings have implications for administering LAIs to first-admission patients.

This study is different from previous studies on administering LAIs among inpatients with schizophrenia in 3 aspects. First, we focused solely on first admitted patients and included a large number of patients nationwide over 14 years rather than any admitted patients of limited numbers from single or few hospitals over 1 to 3 years.^22,23,24,25^ Hence, the prevalence of in-hospital use of LAIs in this study could not be compared directly with that reported in previous studies, which varied substantially, ie, 9% of inpatients from US multistate Medicaid claims,^22^ 8.9% of inpatients at a private hospital in New York City,^23^ 31% of inpatients at a hospital mainly serving a low-income and uninsured population in New York City,^24^ and 13.9% of inpatients in a Switzerland teaching hospital.^25^ Our finding that the prevalence of in-hospital use of LAIs was 16.6% reflected a pattern of underutilization in Asia.^16,17,18^ Second, unlike previous studies that have treated discontinuation of LAIs before 36 weeks as part of outcome (such as those included in a 2019 systematic review by Gentile^21^), this study is the first one that treated early discontinuation of LAIs as a prognostic factor for readmission. Third, we intentionally defined early discontinuation in a way that an inpatient’s early discontinuation of LAIs could be detected at most 2 months after discharge. Based on this definition, we found that not only 60.7% of patients who received LAIs during in-hospital had early discontinuation but also an increased risk for readmission compared with those not receiving LAIs. In contrast, patients who received LAIs during in-hospital without early discontinuation had a decreased risk of readmission.

The correlates of in-hospital use of LAIs provide clues about the characteristics of patients who experienced early discontinuation of treatment. Several correlates, including recent periods and second-generation LAIs, were not associated with the outcome after the exposure was controlled for. Several other factors, including male sex, younger age at first admission, and longer length of stay, remained significant in the multivariable survival regression analysis. These correlations were likely associated with greater clinical severity warranting the use of LAIs. For example, one explanation is that male patients with schizophrenia have a slightly greater incidence of receiving LAIs early in life than female patients are due to men’s earlier age at onset and hence their more severe symptoms.^28,29^

The clinically relevant correlate was patients’ prior antipsychotic use, which might reflect patients’ preadmission illness trajectory and thus remained significant in the multivariable survival regression analysis. For example, for the 21.5% of patients receiving prescription of antipsychotics in each year of the 3-year period prior to admission, they were likely to have undergone antipsychotic treatment for 2 years or longer and thus would be more willing to change to LAIs after being admitted for relapse and had the lowest percentage of early discontinuation. Moreover, this group’s independent association with readmission risk was greatest for patients with the same exposure status due to their longer duration of illness. In contrast, for the 48.0% of patients who were antipsychotic-free during the 3-year period, they were likely to have a first episode and thus least likely to receive LAIs during admission. Furthermore, these patients would have the highest conditional percentage of early discontinuation if an LAI was prescribed. Nevertheless, this group’s independent association with readmission risk was lower than that of the group that had 3 years of prescription due to their shorter duration of illness.

Most importantly, the prognostic validity of early discontinuation of in-hospital use of LAIs was supported by survival regression analyses. When in-hospital use of LAIs was not accompanied by early discontinuation, the readmission risk decreased by 12% (adjusted hazard ratio) or 13% (adjusted time ratio) compared with that of patients not receiving LAIs, similar to the beneficial effect of postdischarge use of LAIs in reducing readmission risk compared with oral risperidone.^16^ However, if in-hospital use of LAIs was accompanied by early discontinuation, the readmission risk increased by 25% (adjusted hazard ratio) or 22% (adjusted time ratio).

Our findings indicate several challenges in promoting early prescription of LAIs for patients with schizophrenia. One challenge is that approximately half of the first-admission patients were antipsychotic-free prior to admission, resulting in patients having little experience with different antipsychotics. This may explain the low proportion of patients who received LAIs and the high percentage of patients who discontinued treatment early. Another challenge is that there is a paucity of research on administering LAIs to first-admission patients, despite the issue of many treatment guidelines.^30,31,32,33^ For example, an expert consensus guideline for LAI treatment in patients with schizophrenia^33^ mentioned that LAI can be considered a first-line medication choice for early stage or even first-episode patients with schizophrenia. However, there is a dearth of studies on how to decrease the incidence of early discontinuation of LAIs. Under these circumstances, LAI-focused staff training might be helpful for enhancing the in-hospital use of LAIs, as exemplified in previous trial studies showing 91.0% in the intervention group vs 51% in the clinician’s choice treatment.^13,34^

### Limitations

This study has several limitations. First, our claims database did not provide information on patients’ clinical parameters. Second, the cohort members of this study were included if they had ever been admitted for treatment. Hence, our results cannot be generalized to patients with schizophrenia who were treated solely in outpatient clinics. Third, there might be residual confounding that were not adequately controlled for in our multivariable analyses because the allocation of first-admission patients to different exposure groups was not randomized in this observational study. For example, the reason why patients who did not receive LAIs had the shortest length of stay at first admission could be either due to the efficacy of the treatment (ie, an outcome) or the attending psychiatrist’s prescription based on patients’ severity of illness (ie, a cause). Future application of target trial emulation^35^ is warranted to assess the causal inference. Fourth, this study neither examined the use of LAIs separately for patients with schizophrenia and patients with schizoaffective disorder, mainly due to the moderate inter-rater reliability^36^ and small number of the latter (approximately one-tenth of the former), nor assessed the influence of comorbidity (eg, substance use disorder) on the use of LAIs, owing to the lack of consistent request or guideline about the listing of comorbid diagnosis in claims data. Nevertheless, we adjusted for the length of stay as a proxy for the treatment difficulties caused by such comorbidity. Finally, we were unable to distinguish whether a patient’s discontinuation of LAIs was due to adverse effects, inadequate efficacy, or nonadherence. Thus, our results were solely based on all-cause discontinuation.

## Conclusions

The prevalence of in-hospital use of LAIs has remained low among patients with a first admission for schizophrenia in Taiwan. In addition to sex, year of admission, prior antipsychotic use, age at first admission, and length of stay, early discontinuation was associated with the risk of readmission, such that early discontinuation was associated with an increased risk and the lack of early discontinuation was associated with a decreased risk compared with treatment with oral antipsychotics alone. Our results have implications for improving the efficacy of administering LAIs to patients with a first admission for schizophrenia.

## Notes

Supplement 1. **eTable 1.** Characteristics of Long-Acting Injectable Antipsychotics Prescribed for First-Admission Schizophrenia Patients Between 2004 and 2017 in Taiwan **eTable 2.** Grouping on the Basis of Each Year’s Antipsychotic Prescription During the 3-Year Period Before the First Admission Among First-Admission Schizophrenia Patients Between 2004 and 2017 in Taiwan **eTable 3.** The Distribution of LAI Prescription Numbers Among Patients Receiving LAIs, by Patients With LAI Early Discontinuation and Without Early Discontinuation **eTable 4.** Sensitivity Analysis by Removing Antipsychotics Use in 3-Year Period Prior to Admission as Covariate in Cox Proportional Hazards (CPH) Model to Assess Potential Collider Effect **eFigure 1.** Generation of LAIs and Proportion of Patients Prescribed LAI During First-Admission by Calendar Years **eFigure 2.** Flowchart for the Cohort Selection of First-Admission Schizophrenia Patients Between 2004 and 2017 in Taiwan and Then Grouped by Whether They Had In-Hospital Use of LAIs and Whether Early Discontinuation of LAI Treatment Occurred Afterward **eFigure 3.** Kaplan-Meier Survival Curves of Patients With Psychotic Readmission Events in the 2004-2017 Cohorts With 3-Level Exposure (LAI With Early Discontinuation, LAI Without Early Discontinuation, and No LAI Prescription) at Admission for a Sensitivity Analysis by Allowing Two or Fewer Continuing LAI Prescriptions Before All-Cause Treatment Discontinuation Occurred

Supplement 2.
Data Sharing Statement
