# Supplementary material for: In-Hospital Use of Long-Acting Injectable Antipsychotics and Readmission Risk in Patients With First-Admission Schizophrenia in Taiwan
Source: JAMA Netw Open. 2024 Jun 17;7(6):e2417006. doi: 10.1001/jamanetworkopen.2024.17006 (PMC11184458; doi:10.1001/jamanetworkopen.2024.17006)
Supplement: Supplement 1. — eTable 1. Characteristics of Long-Acting Injectable Antipsychotics Prescribed for First-Admission Schizophrenia Patients Between 2004 and 2017 in Taiwan eTable 2. Grouping on the Basis of Each Year’s Antipsychotic Prescription During the 3-Year Period Before the First Admission Among First-Admission Schizophrenia Patients Between 2004 and 2017 in Taiwan eTable 3. The Distribution of LAI Prescription Numbers Among Patients Receiving LAIs, by Patients With LAI Early Discontinuation and Without Early Discontinuation eTable 4. Sensitivity Analysis by Removing Antipsychotics Use in 3-Year Period Prior to Admission as Covariate in Cox Proportional Hazards (CPH) Model to Assess Potential Collider Effect eFigure 1. Generation of LAIs and Proportion of Patients Prescribed LAI During First-Admission by Calendar Years eFigure 2. Flowchart for the Cohort Selection of First-Admission Schizophrenia Patients Between 2004 and 2017 in Taiwan and Then Grouped by Whether They Had In-Hospital Use of LAIs and Whether Early Discontinuation of LAI Treatment Occurred Afterward eFigure 3. Kaplan-Meier Survival Curves of Patients With Psychotic Readmission Events in the 2004-2017 Cohorts With 3-Level Exposure (LAI With Early Discontinuation, LAI Without Early Discontinuation, and No LAI Prescription) at Admission for a Sensitivity Analysis by Allowing Two or Fewer Continuing LAI Prescriptions Before All-Cause Treatment Discontinuation Occurred [file jamanetwopen-e2417006-s001.pdf]

Supplemental Online Content

Chen W, Wu C, Liu C, et al. In-hospital use of long-acting injectable antipsychotics and readmission risk in first-admission schizophrenia patients in Taiwan. *JAMA Netw Open.* 2024;7(6):e2417006. doi:10.1001/jamanetworkopen.2024.17006

eTable 1. Characteristics of Long-Acting Injectable Antipsychotics Prescribed for First-Admission Schizophrenia Patients Between 2004 and 2017 in Taiwan.

eTable 2. Grouping on the Basis of Each Year's Antipsychotic Prescription During the 3-Year Period Before the First Admission Among First-Admission Schizophrenia Patients Between 2004 and 2017 in Taiwan

eTable 3. The Distribution of LAI Prescription Numbers Among Patients Receiving LAIs, by Patients With LAI Early Discontinuation and Without Early Discontinuation

eTable 4. Sensitivity Analysis by Removing Antipsychotics Use in 3-Year Period Prior to Admission as Covariate in Cox Proportional Hazards (CPH) Model to Assess Potential Collider Effect

eFigure 1. Generation of LAIs and Proportion of Patients Prescribed LAI During First-Admission by Calendar Years

eFigure 2. Flowchart for the Cohort Selection of First-Admission Schizophrenia Patients Between 2004 and 2017 in Taiwan and Then Grouped by Whether They Had In-Hospital Use of LAIs and Whether Early Discontinuation of LAI Treatment Occurred Afterward

eFigure 3. Kaplan-Meier Survival Curves of Patients With Psychotic Readmission Events in the 2004-2017 Cohorts With 3-Level Exposure (LAI With Early Discontinuation, LAI Without Early Discontinuation, and No LAI Prescription) at Admission for a Sensitivity Analysis by Allowing Two or Fewer Continuing LAI Prescriptions Before All-Cause Treatment Discontinuation Occurred

This supplemental material has been provided by the authors to give readers additional information about their work.

© 2024 Chen W et al. *JAMA Network Open.*

Table S1. Characteristics of long-acting injectable antipsychotics prescribed for first-admission schizophrenia patients between 2004 and 2017 in Taiwan.

Antipsychotic: Generic Name (Brand Name)	Dosage & Forms	Generation	Base	Dose Interval
Flupentixol decanoate (Fluanxol Depot)	20 mg/ml	FGA	Oil	21 days
Fluphenazine decanoate (Flucan Injection)	25 mg/ml	FGA	Oil	21 days
Haloperidol decanoate (U-Dolan Decanoate; Binison; Haldecan; Haldol Decanoas)	50 mg/ml	FGA	Oil	28 days
Clopenthixol decanoate[a] (Clopixol Depot)	200 mg/ml	FGA	Oil	21 days
Zuclopenthixol decanoate[a] (Clopixol Depot Injection)	500 mg/ml	FGA	Oil	21 days
Paliperidone Palmitate[b] (Invega Sustenna)	25, 50, 75, 100, 150 mg pre-filled syringe	SGA	Water	28 days
Paliperidone Palmitate[b] (Invega Trinza)	175, 263, 350, 525 mg pre-filled syringe	SGA	Water	84 days
Aripiprazole monohydrate[c] (Abilify Maintena)	300, 400 mg vial or pre-filled syringe	SGA	Water	28 days
Risperidone (Risperdal Consta)	25 mg, 37.5 mg, 50 mg vial	SGA	Water	14 days

Abbreviations: FGA, first-generation antipsychotics; SGA, second-generation antipsychotics

[a] Clopenthixol decanoate and Zuclopenthixol decanoate were combined in the subsequent analysis due to their small number of prescriptions and similar pharmacological effects.

[b] Paliperidone Palmitate was first introduced to Taiwan in 2012, and first prescribed in first admission period in 2014.

[c] Aripiprazole monohydrate was provided in Taiwan but not prescribed in first-admission period for the patients in this study.

© 2024 Chen W et al. *JAMA Network Open.*

Table S2. Grouping on the basis of each year's antipsychotic prescription during the 3-year period before the first admission among first-admission schizophrenia patients between 2004 and 2017 in Taiwan (N = 56211).

	Antipsychotics in years prior to the first admission					
Group	3rd year	2nd year	1st year	n	n (%)	Remarks
(1) 3 years with prescription	Y	Y	Y	12062	12062 (21.5)	Antipsychotics prescription in each year
(2) Recent yearlong discontinuation	Y	N	N	656	1772 (3.2)	Without antipsychotics prescription in the 1st year before the first admission, but with antipsychotics prescription in earlier years
	N	Y	N	609		
	Y	Y	N	507		
(3) ≤ 2 years with prescription	N	N	Y	10748	15412 (27.4)	With antipsychotics prescription in the 1st year before the first admission, but without antipsychotics prescription in at least one earlier year
	N	Y	Y	3287		
	Y	N	Y	1377		
(4) Antipsychotics-free	N	N	N	26965	26965 (48.0)	No antipsychotics prescription during the 3-year period

Note. If there had any antipsychotic prescription record in the year, it would be marked as "Y". If not, it would be marked as "N".

© 2024 Chen W et al. *JAMA Network Open.*

Table S3. The distribution of LAI prescription numbers among patients receiving LAIs, by patient with LAI early discontinuation and without early discontinuation (N = 9336).

**Number of LAI prescription**	Total (N = 9336)	With early discontinuation (n = 5665)	Without early discontinuation (n = 3671)
Mean (SD)	8.67 (16.34)	1.16 (0.36)	20.27 (21.38)
Minimum	1	1	3
25% Quartile	1	1	5
Median	2	1	11
75% Quartile	8	1	28
Maximum	209	2	209

Note. All of the LAI prescription during first admission period would be counted as once in this calculation. The distribution of prescription numbers was heavily right skewed.

© 2024 Chen W et al. *JAMA Network Open.*

Table S4. Sensitivity analysis by removing “Antipsychotics use in 3-year period prior to admission” from the covariates in Cox proportional hazards (CPH) model to assess potential collider effect (n = 56211).

Variable	Original CPH model: Adjusted Hazard Ratio (95% CI)	Original CPH model: p value	CPH model for sensitivity analysis: Adjusted Hazard Ratio (95% CI)	CPH model for sensitivity analysis: p value
Exposure groups				
No LAI prescription	ref	ref	ref	ref
LAI with early discontinuation	1.25 (1.21, 1.30)	<.0001	1.25 (1.21, 1.30)	<.0001
LAI without early discontinuation	0.88 (0.84, 0.92)	<.0001	0.89 (0.84, 0.93)	<.0001
Sex				
Women	ref	ref	ref	ref
Men	1.03 (1.01, 1.05)	0.017	1.03 (1.01, 1.06)	0.075
Year of admission				
2004-2008	ref	ref	ref	Ref
2009-2012	0.99 (0.96, 1.02)	0.403	0.99 (0.96, 1.01)	0.294
2013-2017	0.94 (0.91, 0.97)	<.0001	0.93 (0.91, 0.96)	<.0001
Antipsychotics use in 3-year period prior to admission				
3 years with prescription	ref	ref	-	-
Recent yearlong discontinuation	0.78 (0.73, 0.84)	<.0001	-	-
≤2 years with prescription	0.87 (0.85, 0.90)	<.0001	-	-
Antipsychotic-free	0.93 (0.90, 0.96)	<.0001	-	-
Age at first admission (years)	0.996 (0.995, 0.997)	<.0001	0.996 (0.995, 0.997)	<.0001
Length of stay of first admission (weeks)	1.014 (1.012, 1.017)	<.0001	1.014 (1.012, 1.016)	<.0001

© 2024 Chen W et al. *JAMA Network Open.*

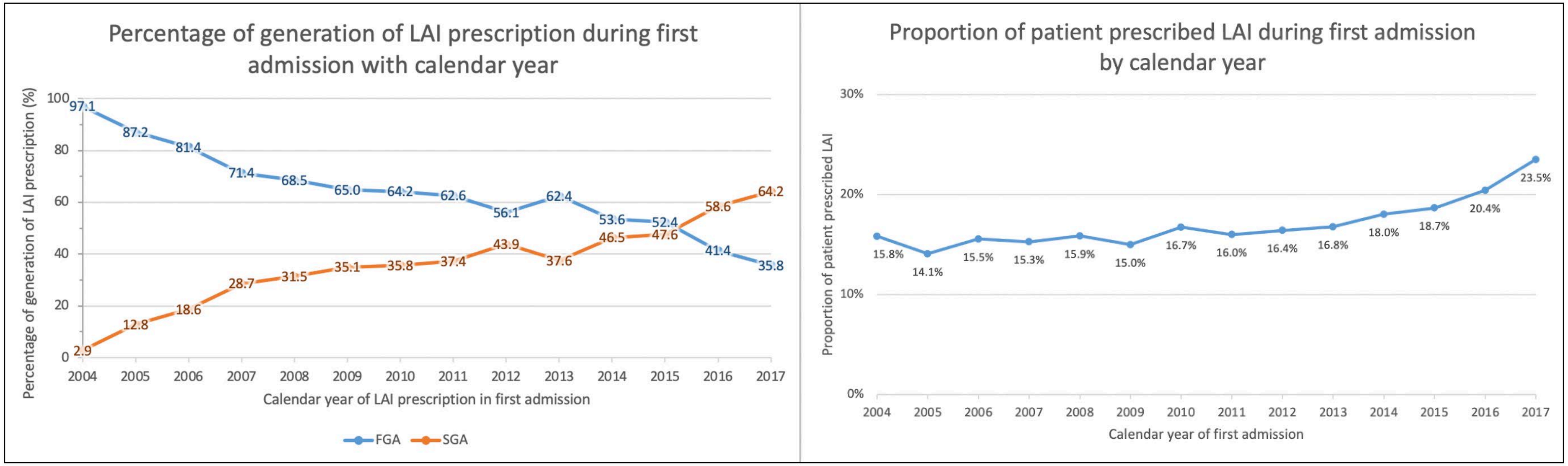

Figure S1. Generation of LAIs and proportion of patients prescribed LAI during first-admission by calendar years.

© 2024 Chen W et al. *JAMA Network Open.*

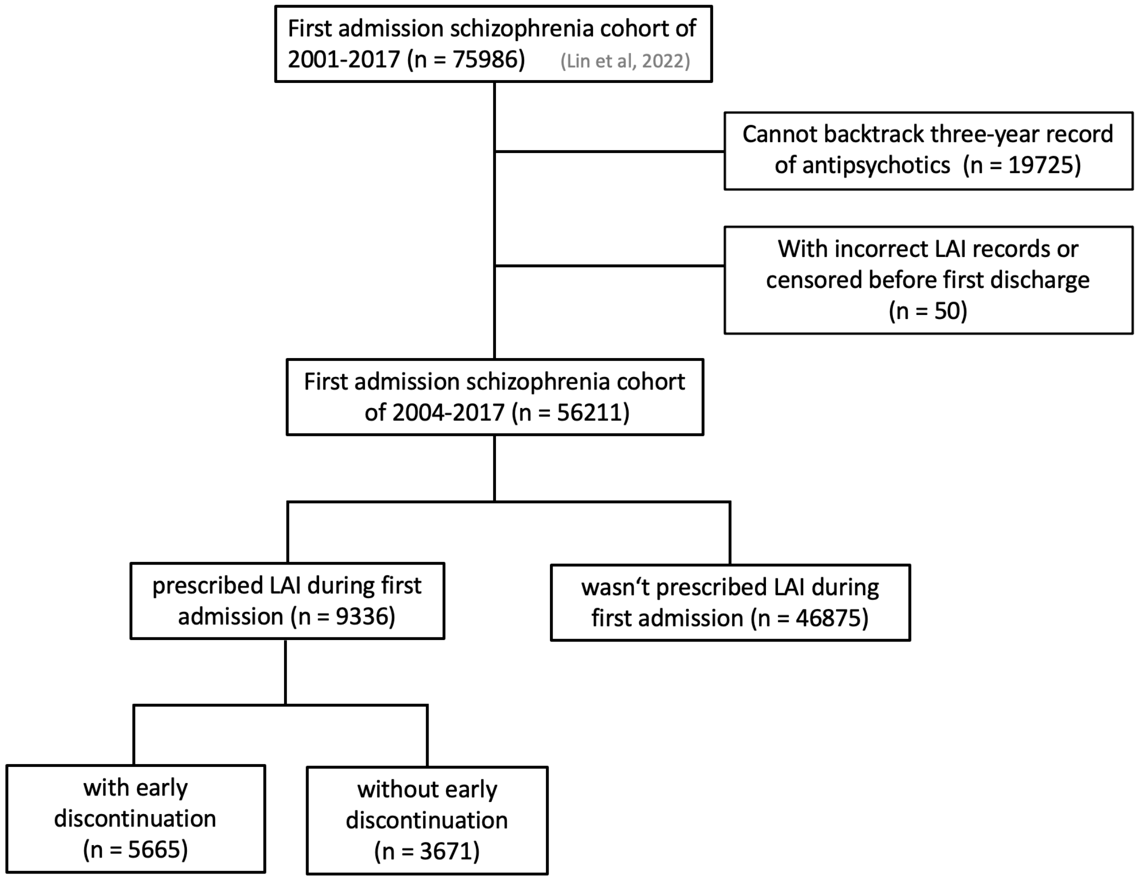

Figure S2. Flowchart for the cohort selection of first-admission schizophrenia patients between 2004 and 2017 in Taiwan and then grouped by whether they had in-hospital use of LAIs and whether early discontinuation of LAI treatment occurred afterward.

© 2024 Chen W et al. *JAMA Network Open.*

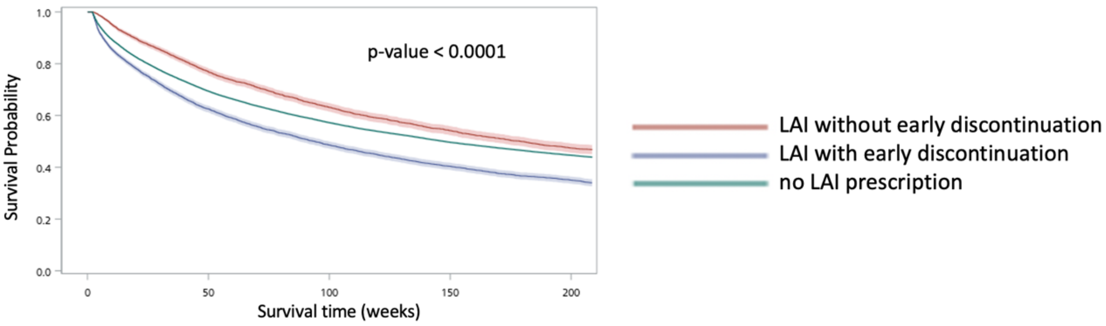

Figure S3. Kaplan-Meier survival curves of patients with psychotic readmission events in the 2004-2017 cohorts with 3-level exposure (LAI with early discontinuation, LAI without early discontinuation, and no LAI prescription) at admission for a sensitivity analysis by allowing two or fewer continuing LAI prescriptions before all-cause treatment discontinuation occurred (n = 56211).

© 2024 Chen W et al. *JAMA Network Open.*
