# Supplementary material for: In-Hospital Use of Long-Acting Injectable Antipsychotics and Readmission Risk in Patients With First-Admission Schizophrenia in Taiwan
Source: JAMA Netw Open. 2024 Jun 17;7(6):e2417006. doi: 10.1001/jamanetworkopen.2024.17006 (PMC11184458; doi:10.1001/jamanetworkopen.2024.17006)
Supplement: Supplement 2. — Data Sharing Statement [file jamanetwopen-e2417006-s002.pdf]

Data Sharing Statement

Chen. In-Hospital Use of Long-Acting Injectable Antipsychotics and Readmission Risk in Patients With First-Admission Schizophrenia in Taiwan. *JAMA Netw Open*. Published June 14, 2024. doi:10.1001/jamanetworkopen.2024.17006

Data
Data available: No

Additional Information
Explanation for why data not available: Data collected for this study are proprietary of the Health and Welfare Data Science Center at Ministry of Health and Welfare, which granted researchers permission and access to the data. The data that support findings of this study are available from these authorities, but restrictions apply to the availability of these data. The code used to analyze these data is available upon request from the corresponding author, for purposes of reproducing the authors.
